# Journeying through Dementia Randomised Controlled Trial of a Psychosocial Intervention for People Living with Early Dementia: Embedded Qualitative Study with Participants, Carers and Interventionists

**DOI:** 10.2147/CIA.S293921

**Published:** 2021-02-04

**Authors:** Kirsty Sprange, Jules Beresford-Dent, Gail Mountain, Ben Thomas, Jessica Wright, Clare Mason, Cindy L Cooper

**Affiliations:** 1 Nottingham Clinical Trials Unit, Faculty of Medicine, University of Nottingham, Nottingham, NG7 2RD, UK; 2 University of Bradford, Bradford West Yorkshire, BD7 1DP, UK; 3 Clinical Trials Research Unit, ScHARR, University of Sheffield, Sheffield, S1 4DP, UK

**Keywords:** psychosocial, self-management, dementia, wellbeing, occupational therapy, qualitative

## Abstract

**Objective:**

To identify the barriers and facilitators to the implementation of a complex psychosocial intervention though a study exploring the experiences of participants, carers and interventionists during a trial.

**Methods:**

Individual semi-structured interviews were conducted with participants, their carers, and interventionists from a sample of recruiting sites that took part in the Journeying through Dementia randomized controlled trial (RCT). Interview data were transcribed and analysed using framework analysis. Co-researcher data analysis workshops were also conducted to explore researcher interpretations of the data through the lens of those with lived experience of dementia. Triangulation enabled comparison of findings from the interviews with findings from the co-researcher workshops.

**Results:**

Three main themes emerged from the interview data: being prepared; intervention engagement; and participation and outcomes from engagement. From these themes, a number of factors that can moderate delivery and receipt of the intervention as intended were identified. These were context and environment; readiness, training, skills and competencies of the workforce; identifying meaningful participation and relationships.

**Conclusion:**

This study highlighted that the observed benefit of the intervention was nuanced for each individual. Mechanisms of change were influenced by a range of individual, social and contextual factors. Future research should therefore consider how best to identify and measure the multifaceted interplay of mechanisms of change in complex interventions.

**Trial Registration:**

ISRCTN17993825.

## Background

Approximately 537,000 people are living with a diagnosis of dementia in the UK^1^ of which around 55% are in the mild stages of the condition.^2^ Both global and UK policy recommendations emphasise the importance of post-diagnostic treatment and support to help people live well with a diagnosis of dementia.^3^,^4^ Guidance in the UK now stipulates that activities to promote wellbeing should include psychosocial and occupational therapy interventions.^5^ Proposed benefits include the promotion of self-efficacy to support independent living and meaningful activity.^6^

Self-management interventions are an established approach for those living with long-term conditions and are supported by the UK and international policy.^7^ Participants are enabled to identify strategies to take back control and responsibility for their health and wellbeing. There is growing evidence for use of self-management techniques with people living with dementia; however, there is still a lack of high-quality research to examine the effectiveness of such interventions.

In complex interventions, understanding the^7^,^8^ mechanisms of action and how they interact is challenging. The multi-component nature of such interventions means that any one part or the combination of parts may be a factor for change. It is important therefore that each individual component is described and evaluated.^9^,^10^ MRC guidance recommends integration of qualitative methods to explore the interaction between mechanisms of change, context and outcomes.^11^

Journeying through dementia is a manualised occupational therapy-based self-management complex intervention for those with mild dementia and was co-created with people with lived experience of the condition.^12^,^13^ The intervention is underpinned by social cognitive theory,^14^ with a focus on behaviour change through increased self-efficacy^15^ and effective problem solving thereby promoting increased self-management, independence, improved wellbeing and life satisfaction. It acknowledges the dynamic transactional relationship between the person, the environment in which they live and their occupations and roles.^16^

The intervention was evaluated through a pragmatic, two-arm, parallel group, individually randomised controlled trial.^17^ A total of 480 participants were randomised of which 241 received the intervention. The intervention consisted of 12 consecutive facilitated weekly group meetings attended by up to 12 people held in the community. Participants also received four one-to-one sessions with a facilitator to focus on personal goals. During group sessions, participants were encouraged to explore topics from the manual using discussion and activities including community engagement. Consolidation of learning and experience was aimed for through a minimum of three out of venue activities (otherwise referred to as outings). Intervention facilitators received 2-days' training before commencing intervention delivery and had regular supervision from a senior clinician at their employing site throughout the period of the intervention delivery.

To better understand how a novel intervention such as Journeying through Dementia might be applied in a real-world context it is essential to ask a wide range of stakeholders involved in delivery and receipt for their experience, opinions and ideas.^18^ Involving people with lived experience of a condition as co-researchers in meaningful co-creation of data through interpretation of findings by working alongside academics is becoming more accepted and respected.^19^ Co-research adds validity by bringing insight to the data from a unique perspective of those with the condition.^20^ This enables the identification of commonalities and divergences between researcher interpretations of the data is commensurate with those of people with lived experience of dementia. It has been demonstrated that it is possible and can be productive to involve people with lived experience of dementia as co-researchers in qualitative analysis.^21^

This paper reports a qualitative sub-study, embedded within a trial, which explored the perspectives from those involved in delivering and receiving Journeying through Dementia. Thereby evaluating how the intervention produces change and to inform future development of the intervention.

Reporting meets the requirements of COREQ guidance.^22^

## Aims and Objectives

The main aims were to understand the mechanisms of a complex psychosocial intervention and to identify the barriers and facilitators to implementation, through an exploration of the experiences and insights of interventionists (those facilitating the intervention and those supervising the facilitators), as well as those in receipt of Journeying through Dementia. This included

- The range and nature of issues that influence the experience of the intervention.
- Factors that may mediate or moderate the effectiveness of the intervention.
- Perceived skills and competencies required to facilitate the intervention.
- Effect of the intervention on participation and living with the diagnosis.

## Materials and Methods

We conducted interviews with people living with dementia who had attended the intervention and their participating carers (if they had been recruited to the study) as well as with interventionists (all were NHS staff). We also interviewed participants who had withdrawn from the intervention to obtain different perspectives. The analysis was also informed by co-researcher data interpretation workshops which were convened with people with lived experience of dementia.

### Sample

The Research and Development (R&D) teams and Principle Investigators, at a purposive sample of four (out of 13) NHS delivery sites participating in the Journeying through Dementia trial, were approached to take part in the qualitative sub-study and all agreed. Site selection was pragmatic with criteria including approvals being in place, that sites were open and running the intervention, sites had or planned to conduct at least two successive intervention programmes for the trial and that interventionists had the capacity to participate in interviews. Consideration was also given to geographical location and population size so that selected sites represented the range of those that participated in the trial.

Intervention facilitators were sought from any relevant UK NHS professional background working at band 4 on the UK NHS Agenda for Change (AfC) pay scale.^23^ In reality, we recruited facilitators working across bands 2–8 on the AfC pay scale. Supervisors were experienced clinicians from a range of clinical backgrounds employed at AfC bands 6–8.

A sampling frame (see Table 1) was used to purposively identify participants at each selected site who had received the intervention during a second wave of intervention delivery including participants who had withdrawn from the intervention. We also sought to interview the carers of participants who were being interviewed (see Table 2). Interviewed participants were selected to be representative of the overall trial population. Of those interviewed only age was not representative of the overall trial population but was representative of those who took part in the groups included in the qualitative sub-study. A total of 19 participants and 14 carers were invited to take part in the interviews, with 15 participants and 10 carers subsequently accepting. The spread of participants and carers approached and agreed to take part in interviews across the four sites is presented in Table 3.

**Table 1:** Participant Characteristics

Characteristics	Participant (n=15)
**Diagnosis**	Alzheimer’s, n=6
	Frontotemporal, n=2
	Vascular, n=3
	Mixed, n=3
	Other – mild dementia, n=1
**Age**	Range, 67 –90
	Average, 77
**Ethnicity**	White British, n=14
	African/Asian, n=1
**Gender**	Female, n=7
	Male, n=8
**MMSE score**	Range, 20–29
	Average, 26
**Living arrangements**	Living alone, n=2
	Living with others, n=13

**Table 2:** Participating Carer Characteristics

Characteristic	Participating Carer (n=10)
**Age**	Range, 50–89
	Average, 71
**Gender**	Female, n=7
	Male, n=3
**Relationship**	Spouse, n=9 Child, n=1

**Table 3:** Spread of Participant Interviews by Participating Site and How They Were Conducted

	Total Number Approached		Declines		Number of Individual Interviews		Number of Joint^†^ Interviews
**Site**	**Participants**	**Carers**	**Participants**	**Carers**	**Participants**	**Carers**	
1	6	5	1	1	3	2	2
2	5	5	2	2	3	3	0
3	3	1	0	0	3	1	0
4	5	3	1	1	2	0	2
**Total**	**19**	**14**	**4**	**4**	**11**	**6**	**4**

**Note:** ^†^Where the participant and carer were interviewed together.

Of the interventionists, all facilitators (n=10) and four of the five supervisors from the four selected sites were interviewed. All staff were employed in the participating NHS organisations.

### Materials and Tools

Interview information sheets, consent forms and appointment letters were created by the research team; one version for participants and their carers and one for the interventionists. The trial Advisory Group of people living with dementia reviewed and advised on acceptability, content, language and presentation of the participant and carer versions of the documentation. They also suggested including a photograph of the researcher in the appointment letter to support participant recognition and reassurance on the day of the interview.

Interview topic guides (see supplementary documents) were initially developed by the qualitative lead for the trial using tacit knowledge and best evidence; with other members of the research team then contributing towards refinement of the tools. For the participant and carer interviews, enhanced methods of communication were employed to support engagement and try and ensure meaningful findings.^24^,^25^ The trial Advisory Group reviewed the topic guides and intended interview process with study participants and carers for usability and acceptability. Following their advice, we amended the language in topic guides, reduced the length of the proposed interview and implemented suggestions to aid recall such as having the intervention manual at the interview for participants to refer to.

### Semi-Structured Interviews

Individual semi-structured interviews were conducted with participants and carers as well as interventionists from the four participating sites. The number of interviews achieved met those aimed for in the trial protocol.^17^ Interviews were conducted post completion of a second intervention group at each site. This was to allow interventionists time to become familiar with Journeying through Dementia so that when interviewed they could refer to delivery as intended rather than the learning period that would have occurred when implementing a new intervention.^26^ All those who agreed to take part were free to withdraw at any time.

Interviews were conducted by three experienced members of the research team including the lead for the qualitative study (KS) and two researchers (JBD and BT).

Interviewers re-confirmed consent prior to commencement of the interview. Permission was sought to audio record each interview and all interviewees consented to recording. All recordings were transcribed and copies of individual transcripts shared with the interviewee for respondent checking to ensure what they had said had been intended. This was also considered a step to support participant engagement through scaffolding memory for consent for quotation to be used.^27^ Transcripts were sent by post to all those interviewed accompanied by a letter providing details of what to do and how to contact the study team should they have any further comment. Two of those interviewed (both facilitators) provided additional comment on their transcripts using annotation and one of these also altered some of the grammar (interviews were transcribed verbatim).

#### Participant and Carer Interviews

Consent was obtained to contact participants and carers to take part in interviews. The intervention facilitators provided a verbal reminder to participants about the potential interviews during the last few group sessions. The interview sampling frame was then created to select interviewees from those who had consented and approaches to those subsequently identified were made by telephone as soon as possible after the end of intervention delivery. Due to delays obtaining relevant approvals, confirming interviewee availability and changes to research staff, there was a time lag between finishing the intervention and the interview for some interviewees which may have contributed to poor recall. During the telephone call to finalise consent and make necessary arrangements, the researcher explained the purpose of the interview and what would happen. They emphasised the points that the participant or carer could decline to take part, they should take time to consider participation, and they could agree to participate verbally. If they declined, no further contact was made regarding the interviews. If more time was requested to consider their participation, they were sent a copy of the information sheet and a time was agreed and arranged for a follow-up contact. If they agreed to be interviewed an appointment was made and they were sent a copy of the information sheet and a personalised letter confirming the interview appointment details. A copy of participant interview arrangements was also sent to their carer if the participant requested this.

A further telephone call was made to the participant (or their carer if requested by the participant) prior to the interview as a reminder. Interviews occurred between 6 March and 19 July 2018 taking an average of 38 minutes (range 13–75 mins) and all were conducted face-to-face in participants’ own homes.

Individual participant interviews were encouraged to provide a confidential space for each voice to be heard and of the 15 interviews with people with dementia, 11 were achieved individually. However, it was the participants' decision to have a carer present or not. Nine individual interviews occurred in another room, but due to space limitations, two took place with the carer present in the same space. Where dyad interviews (n=4) were conducted this was at the request of the participant. Reasons for requesting a joint interview included participant reassurance or couples expressing they were a “partnership”.

#### Interventionist Interviews

An invitation was sent to individual facilitators and supervisors via email including an information sheet and consent form. Those who agreed to take part then contacted the research team and an appointment was arranged. Consent was obtained via email as all interviews were conducted by telephone. Interviews occurred between 30 Nov 2017 and 5 April 2018 and were an average duration of 49 minutes (range 30–70 mins).

### Co-Researcher Workshops

Two co-researcher data analysis workshops were held with people living with dementia and their carers acting as co-researchers. Co-research is a method of research enabling participants to act as joint contributors alongside academics.^28^ Benefits include bringing insight to the data from a unique perspective of those with the condition. This enables identification of commonalities and divergences between researcher interpretations of the data and those of people with lived experience of dementia. Two workshops were organised to allow coverage of a reasonable amount of data and limit the length of each session so that those participating did not fatigue.

Our co-researchers were recruited from the Journeying through Dementia trial Advisory Group and from the Bradford Experts by Experience cohort https://www.bradford.ac.uk/dementia/experts-by-experience/. The first workshop was attended by seven co-researchers (two married couples in which one person had dementia and the other was their spouse and carer, one current carer, one past carer and one independent person living with dementia) and the second was attended by 12 co-researchers (five couples in which one person had dementia and the other was their spouse and carer, one independent person living with dementia and one independent carer). Three people (two people with dementia and one carer) attended both sessions. The workshops were hosted in community venues and remuneration for co-researchers followed INVOLVE best practice guidelines.^29^

Each workshop was led by two members of the research team, supported by other team members who took written notes of the discourse and any other salient observations. Written consent to audio record each session was obtained from each participant at the outset to supplement note taking and observations. Both workshops took place in the early stages of the data analysis process to check researcher understanding and interpretation of the data before the final classification framework was produced. Involvement of two researchers and the co-researchers aimed to reduce bias to any one point of view.^30^ A comprehensive account of the process and outputs from the co-researcher workshops will be provided in a separate publication.

### Data Analysis

#### Theoretical Perspective

The theoretical lens of critical realism assumes that not all knowledge is equal when trying to understand the real world but that there are multiple ‘truths’ that need to be acknowledged in order to explain social constructs.^31^ By exploring a range of stakeholder perspectives (participants, carers and interventionists) we aimed to obtain the range of “truths” as perceived by different stakeholders and individuals and therefore provide a detailed and nuanced understanding of the implementation and receipt of the Journeying through Dementia intervention.

#### Methods of Analysis

Analysis of the data was conducted by the qualitative lead and two researchers. The interview topic guides were used to provide an initial framework for the analysis, but coding was also inductive to holistically understand the data. This iterative process allowed for collapsing or expanding of initial themes and sub-categories. Coding was initially undertaken separately for participants and carers from the facilitators and supervisors before merging to produce a final classification framework. This approach enabled the coders to manage the quantity of data as well as allow varying perspectives to emerge from the data.

Framework analysis^32^ was used to explore the range of perspectives (multiple truths) in the interview data provided by participants, carers, facilitators and supervisors as required for critical realism.^31^ Resulting themes identified from the data were evidenced by quotation from the interview transcripts. This method provides a clear step-by-step approach to analyse large data sets as described below, can link topics within the analysis, enables a holistic overview from multiple sources, takes into account the perspectives of more than one researcher and addressed the research questions. Data were managed using NVivo 12 computer-assisted qualitative data analysis software (CAQDAS).

#### Step 1 – Familiarisation and Independent Open Coding

All transcripts were read for data familiarisation by two researchers who then independently open coded a purposive sample of half of the transcripts. The transcripts were selected to represent all four fidelity sites, the different interviewers and a cross section of interviewee viewpoints reflecting both positive and negative experiences of taking part.

#### Step 2 – Development of Initial Frameworks

The two researchers then reviewed the open coding together to create an initial framework for analysis of the participant and carer interview data. The same process was followed for the second framework for analysis of the facilitator and supervisor data to ensure that different perspectives were maintained.

#### Step 3 – Co-Researcher Review of Participant and Carer Interview Data

A selection of quotes illustrative of the range of themes and sub-categories identified from the initial framework (Step 1) created out of the participant and carer interviews were then presented at our co-researcher analysis workshops. A total of 20 quotations (9 in workshop one, and 11 in workshop two) were reviewed by the group. Each quotation was presented to the group one at a time typed in large font in black text on yellow paper^25^ ensuring text was easier to read for those with compromised vision and/or sensory impairments. The co-researchers were then given time to read the quotation as well as hearing the workshop lead read it out loud. They were asked to review and respond to the quotation with particular focus upon language (what was being said and the words used), for example, was the content positive, negative or ambiguous? We also asked them to consider tone, for example, strength of the voice; was it passionate or dispassionate and finally we requested consideration of the overall meaning of the quote; that is their interpretation of what was being said. For each quote, the group was made aware of the voice being used ie participant or carer and were given a brief context, ie talking about an individual session or a group meeting. Interpretation of each quotation discussed by the co-researchers was tabulated with that of the researchers’ understanding and compared for convergence, divergence or novel interpretation. Where interpretations diverged or were novel these were subsequently reviewed by the two researchers. Interpretations were used to develop the initial participant and carer framework produced in Step 2.

#### Step 4 – Application of Frameworks to All Transcripts and Development of Modified Frameworks

The two researchers applied the initial frameworks to code all the transcripts and then reviewed the coding together to identify any new emerging themes. This resulted in two modified frameworks, one for participant and carer data and a second for facilitator and supervisor data.

#### Step 5 – Comparison and Merging of Modified Frameworks

The two modified frameworks were then merged to compare and contrast perspectives and to chart the data into a matrix producing a final classification framework identifying key themes across all the data combined.

#### Triangulation of Qualitative Findings

Triangulation can increase the validity of research findings by combining and evaluating multiple data sources and data collection methods.^33^ A data triangulation protocol (see Table 4) was created and applied to enable comparison of the multiple data sources (range of interviews from different sources and interpretations from the workshops) and reduce the risk of bias.^34^

**Table 4:** Triangulation Protocol for Qualitative Analysis

Step	Activity	Source
1. Sorting	Sort the findings from each data source under the final classification framework to determine where there maybe overlap or divergence, presence, frequency and meaning.	Interview transcripts with participants, carers, facilitators and supervisors
2. Convergence coding	Identify key themes from each data source using coded examples to determine prominence and compare findings. Then compare a sample of coded examples with co-researcher interpretations from the perspective of people living with dementia.	Interview transcripts with participants, carers, facilitators and supervisors. Co-researcher workshop selected quotation.
3. Convergence agreement	Identify where there is agreement, divergent or ambivalent themes and interpretations between the two coding researchers and the researchers and co-researchers (people living with dementia).	Initial and modified analysis frameworks. Co-researcher workshop selected quotation.
4. Convergence assessment	Identify global assessment for level of agreement between multiple researchers (including the co-researchers) as well as acknowledging different perspectives.	Final classification framework.
5. Completeness	How results of the global assessment enhance the completeness of the overall findings and answer the research question.	Final classification framework.

## Findings

Three main themes were identified from the analysis, see Table 5. Unique identifiers are used for reporting direct quotes and confidentiality maintained by removing identifiable information. Quotations have been selected to illustrate the identified themes.

**Table 5:** Themes Identified from Interview Data

1. Being Prepared	2. Intervention Engagement and Participation	3. Outcomes from Engagement
Intervention training Resources and time commitment	Eligibility Group dynamics Out of venue activities (outings) One-to-one sessions Intervention facilitation	Confidence through knowledge and gaining perspective Enjoyment, feeling valued or empowered Social contact and friendships

### Theme 1 – Being Prepared

#### Intervention Training for Interventionists

The opportunity to practice components and share learning during the training was highlighted by all interviewed interventionists as being particularly helpful. However, several facilitators, including those who stated appreciation of the practical exercises during training, also described how the training had not met all their needs.

I don’t know whether it was just because there was quite a lot of information over the two days … we came away just a little bit confused. (Facilitator H)

#### Resources and Time Commitment

Most interventionists indicated that, over time, experience of delivering the intervention led to greater proficiency. Some facilitators found the manual a helpful “toolbox” to dip in to whilst others thought it was overly comprehensive. One facilitator who said that the manual was too comprehensive also considered that the contents were therefore not always appropriate for people with more advanced impairments.

We had a couple [of participants] who were quite further on in their [dementia] journey and so I guess lots of the materials and lots of the discussion points and activities were really extremely difficult for them to do and engage in. (Facilitator G)

The size of a group, the needs of the individual participants and the type of activities that the group engaged in were all cited by facilitators as factors that could influence resource requirements as illustrated by the following quote;

Three [staff] as a minimum, but equally sometimes three was difficult particularly when we were going on outings. When we went on outings I felt that we needed probably at least four depending on the outing really. (Facilitator F)

All facilitators considered that the estimated time to undertake all aspects of the intervention (1 day per week) was inadequate but that the required time did diminish over time. However, it had still remained in excess of 1 day.

… we had a bit of insight into what had worked for that first group and what had not … So yes it took less time second time around definitely. However, it still took a lot of time … (Facilitator B)

### Theme 2 – Intervention Engagement and Participation

#### Eligibility

All interviewed interventionists considered that the wide-ranging physical and cognitive impairments experienced by people with dementia had not matched their expectations and had challenged their ability to plan, organise and deliver the intervention as well as enable meaningful involvement of participants.

… I thought we were going to end up with people who were newly diagnosed and quite cognitively intact in those with mild dementia. That’s not how it was. (Facilitator B)

Examples of how attempting to meet the needs of the whole group could lessen the experience of individual participants were provided;

There was still a couple of participants who were restless because they wanted to do more but we were almost having to tailor it for the slowest person. (Facilitator F)

Conversely, reports of participants continuing activities after the intervention had ceased suggested that at least some of those who had taken part were able to apply their experiences in their lives going forwards.

#### Group Dynamics

Participants, carers and interventionists spoke about the impact of group dynamics and diversity factors that could influence their experiences of delivering or receiving the intervention.

A smaller group for me it’s important, if you have a larger group, people may not share so much … ” (Participant L, Individual)

It was useful because in fact although there were one or two men there similar to myself … you do meet people that are not within your same sphere. (Participant D, Individual)

Participants expressed varying perceptions and requirements of the intervention. Consensus obtained from one of the co-researcher workshops was that this quotation discussing the importance of the intervention setting was about respecting the needs and feelings of those taking part. Privacy, including in some instances from their carers was important for participants when discussing personal issues.

You also need a setting that is private because of the sensitive nature of the subject … it has to be in a place where we all knew it was safer, rather than public. (Participant L, Individual and quotation explored in the co-researcher workshop)

Having common ground was important but many of those interviewed (interventionists and participants) acknowledged that meeting people with different experiences was important for sharing and learning.

It did a terrific amount of good to you, to find that there was other people, you were not the only one …. (Participant E, Individual)

I think there was a few people within the group [who] reflected on peoples’ different ways of approaching things. And if there hadn’t been the group they would have never seen life in that different perspective. (Supervisor A)

#### Out of Venue Activities (Outings)

Accompanying people with dementia into the community caused concern for some interventionists who expressed worries about the risks involved and the organisational accountability for this. There appeared to be tensions between duty of care to patients versus the “self-management” ethos of the intervention.

We are professionals; we are entering into this with service users. We have a responsibility and a duty of care. We need to do things properly. We do need to risk assess because we’re in paid employment and we work for an organisation that would expect that of us … (Supervisor B)

Interestingly, despite concerns from some interventionists regarding the complexity of the intervention and in particular how participant ability could be challenged, some facilitators and most participants talked positively about the most challenging component of the intervention, the outings.

To be able to have the freedom to go out and about into the community with people, again that is something that we don’t ordinarily get to do within our service. (Facilitator D)

One facilitator described how they had investigated activities for potential risk prior to suggesting them and also using the first outing as a test of abilities of the group. This person considered that selection of, and expectations of the outings had to be carefully managed due to the physical, as well as cognitive, abilities of group members.

Because of the level of physical problems … it limited the range of the outings … until we got people out of there (in-venue) we didn’t realise how impaired they were both cognitively and physically. (Facilitator B)

#### One-to-One Sessions

Although the majority of facilitators recognised the importance of the one-to-one sessions, particularly for individual goal setting, all interventionists (facilitators and supervisors) described this activity as challenging, citing lack of participant understanding of the purpose of such sessions and lack of participant engagement.

I didn’t get a goal from anyone, I mean most of them said I just want to see what it’s [intervention] all about, which isn’t a goal … (Facilitator F)

Sometimes it felt like I was trying to force a goal on them. (Facilitator A)

However, other facilitators described how goals could be identified and explored if participants were given time and support.

Quite a few participants have struggled to know what goals they would like to achieve … I have just reassured them … and on subsequent sessions, we’ve been able to nurture those ideas into tangible goals. (Facilitator D)

Participants who recalled taking part in one-to-one sessions described them as being an opportunity for personal time and identifying support to meet their needs.

I valued those one-to-one sessions quite a lot because that was ‘me’ time with [facilitator] … because I knew that was just me and her talking about the things that I felt uneasy about or something that I felt strongly about, so to me they were quite valuable sessions. (Participant L, Individual)

#### Intervention Facilitation

Positive relationships between interventionists (co-facilitators and supervisors and facilitators) and between participants and facilitators were all described as being vital for successful intervention delivery and promoting the engagement of all taking part.

We’ve [co-facilitators] got a good working relationship … we recognise each other’s skill set and what’s worked well in previous groups. So we will bring those skills together. (Facilitator D)

[Facilitators] were brilliant, they let you talk, they shut you up when they had to … they didn’t tell you what to do, they guided you, signposted you, they were brilliant. Yes I didn’t feel as if they were patronising me in any way. (Participant K, Dyad)

Compared to existing service provision, Journeying through Dementia was described by several interventionists as being more proactive and interactive, offering early professional input, a community focus and supporting enactment through the outings.

I’m working in a situation we haven’t got OTs [Occupational Therapists] in at that earlier stage of someone’s journey and I really see the significance and the value. So for me it’s really reminded me of what difference some of this work can make on peoples’ journey in life and to be able to take some control over their own situation and what benefit that is. (Supervisor A)

Several facilitators were of the view that without carer involvement some participants, and in particular those with more severe cognitive or physical impairments, could struggle to take part in the intervention.

Where they [participant] are by themselves with no carer it’s a very different picture both on the one-to-ones and … attendance to the sessions. (Facilitator B)

I think it’s the ones where the carers are keen in this group, they might keep in touch but if the carers aren’t then they might not. (Facilitator A)

Conversely, some interventionists also expressed that greater carer engagement with the intervention could change the balance of power, thereby disadvantaging people with dementia.

… I think they [participants] need that independence when they come to the group sessions. Sometimes having the carers there can stop them from saying things because they probably don’t want to upset them. It would be difficult for them to be more open about their experiences. (Facilitator E)

### Theme 3 – Outcomes from Engagement

Despite the impact of impairment upon ability to take part many of the interviewed participants, although not all, reported a range of good outcomes and described positive behaviour change as a result of attending the intervention.

#### Confidence Through Knowledge and Gaining Perspective

Most participants talked about gaining in confidence due to their involvement. Our co-researchers felt the following quote was a positive statement which revealed how this participant had gained confidence by focussing on what they could still do.

It [the group] wasn’t all about how bad my situation is, I’m going to lose my memory, I’m just going to lose it – No it was just a good thing, it was kind of building confidence not destroying people, but building them up. That there is life after this. (Participant L, Individual and quotation explored in the co-researcher workshop)

Group dynamics through sharing and peer support also played a significant role in supporting engagement in meaningful activity and enabling behaviour change. Our co-researchers were in agreement with the overall meaning of the quote but also focussed on the nuances of choice. On diagnosis people with dementia usually experience loss of self-management and decision-making, here the co-researchers considered that the individual had taken back control supported by the group.

I’ve learned how to swim again. I didn’t have the confidence to swim …. Well it feels like you’ve achieved something again, something worthwhile. I thought these groups was going to be all sort of bingo and nostalgia … but when I went to the group and I met these people and they weren’t just talking … they were doing these things. (Participant K, Individual and quotation explored in the co-researcher workshop)

The majority of participants described valuing the opportunity to share their experiences and learn from others in the same situation. This was reiterated by our co-researchers who commented those newly diagnosed typically do not get a lot of information from health professionals. They agreed that confidence came with knowledge.

I think I got more information about the difference between Alzheimer’s and Dementia … and it was interesting listening to the other people as well, how they felt about their diagnoses and how people reacted to it. (Participant L, Individual and quotation explored in the co-researcher workshop)

#### Enjoyment, Feeling Valued or Empowered

The majority of interviewed participants described enjoying the intervention, and particularly the new experiences it introduced to them. Only two expressed disappointment but had continued to attend intervention sessions.

I thought it was quite a flippant intervention and I expected it to be a bit more digging deep and it didn’t do that (Participant M, Individual)

Several participants talked about how attending the intervention had enabled them to contribute toward their community. For example, one participant started volunteering at the local Alzheimer’s Society. Another expressed how they had become more independent.

It’s making me a bit more doing it myself not just sitting in and letting everybody else do it. (Participant I, Individual)

This was also observed by several carers.

… having done that group he then decided he would go to respite care for a day … it made him decide that he would go somewhere, that was the really positive thing that came out of it. (Carer A, Individual)

#### Social Contact and Friendships

Nearly all the participants talked about how the intervention had promoted social contact and friendships both during and post intervention delivery. However, participants also described the immediate sense of loss of social contact or friendships once their involvement ceased. This was despite being advised and supported by the facilitators regarding how they might keep in touch with others. Fear of rejection, memory, logistics or need for someone to take responsibility for facilitating communication was cited by various participants as reasons for not meeting with others after intervention cessation.

I thought well I’m sitting here saying nobody’s [contacted] me, but I haven’t done it to anybody else either … it’s a different environment though isn’t it, I think you feel safe if you’re in a group, rather than [contacting] somebody and perhaps get a negative [response]. (Participant O, Dyad)

## Discussion

The goal of the intervention is to enable the knowledge and skills to promote self-management and support independence. Achievement of this leads to improved wellbeing for the individual. The significant factor we identified that appeared to mediate behaviour change was the dynamic transactional relationship between the person, the environment in which they live and their occupations and roles. This meant that reported benefits were nuanced in relation to impact and outcomes. A number of factors also emerged as being moderators of delivery and receipt of the intervention as intended. These included context and environment; readiness, training, skills and competencies of the workforce; and identifying meaningful participation and relationships.

### Context and Environment

The community based, participant-led self-management ethos of the Journeying through Dementia intervention did not always fit well within the organisational (NHS) or professional approach to risk management and patient safety. Current interventions tend to be limited to short-term programmes conducted within NHS premises or involve referral to the voluntary sector. The comfort and familiarity of a setting or workplace may be preferred by those delivering novel interventions and being able to fall back on organisational processes provides a safety net. Working in different ways to deliver Journeying through Dementia did present a challenge to some interventionists. Delivery within a health setting automatically distances an intervention from everyday life for those taking part and is particularly problematic when the intervention is community focussed.

### Readiness, Training, Skills and Competencies of the Workforce

In comparison to interventions currently available within NHS services, Journeying through Dementia was described by interventionists as being time consuming and resource hungry due to its length and multi-layered complexity. Whether organisations like the NHS should allocate limited resources to the intervention would need to be considered against the population-based impact of the intervention. This raises several questions. Is there a desire at the organisational level within UK health services to offer person-centred community-based complex interventions like Journeying through Dementia as part of everyday care provision? And secondly, whether the NHS has the resources to deliver such interventions safely to the volume of people needing support. How best to support people to live well with dementia in the community remains unresolved without further investment not only in research but also in in-service provision.

### Identifying Meaningful Participation

Readiness to engage in interventions and meaningful occupation not only refers to service provision but also a need and desire to engage from participants and their carers.^35^ The impact of impairment on participation in occupational activity is complex and observations of capacity and capability may be influenced by other factors such as the individuals resilience,^36^ self-confidence or self-determination^37^ all of which are reliant on an intact and integrated sense of self.^38^ Immediately post diagnosis is when many people will experience uncertainty and doubt about their future.^39^ This intervention therefore is not just about adaptation of the activities to meet the needs of each individual it is also about challenging these uncertainties to provide a way forward for people living with dementia to live well with their diagnosis.^39^ Despite participants having only mild dementia according to assessment criteria, it was still considered by the interventionists that for those with greater functional memory deficits the impact of the intervention would be limited. Impairment was overcome for some participants through the combination of intervention content, group dynamics, individual resilience and facilitation which was found to bolster participant confidence and support learning of methods of compensating for impairment. Therefore, who might benefit from this intervention cannot be determined by cognition alone.

### Relationships

The complexity of relationship dynamics and particularly the social networks of individuals was difficult to capture and understand in the trial setting.^40^ However, the diversity and opportunity for social contact within groups was perceived as a strength. In addition, a sense of reassurance through commonality and acceptance was located in the group aspect of the intervention which supported participants to maintain this contact and group cohesion during delivery. However, participants and their carers clearly indicated the need for continued contact and social engagement. Since an ongoing intervention would not be possible within a health-care setting due to limited resources,^41^ support is focussed on organisations in the UK such as the Dementia Engagement and Empowerment Project (DEEP)^25^ who support people living with dementia in the community. However, the question remains whether they could also help provide a longer-term solution for interventions like Journeying through Dementia.

The qualitative results will provide deeper insight into the findings of the RCT which only provide a view from a whole trial population level. The qualitative results allow us to identify which aspects of the intervention were acceptable and where there were challenges to delivery in the NHS. These issues would not have been evident from the trial analysis alone.

## Conclusion

This study highlighted that any observed benefit of the Journeying through Dementia intervention was nuanced to each individual. A one size fits all approach to intervention design cannot accommodate the complexity of dementia and people’s experiences of living with dementia. Post-diagnostic care should be holistic, incorporating social care, physical and mental health.^42^ As people are living longer and the number of people living with dementia as well as other co-morbidities increases, complex community-based interventions to support their health and wellbeing seem inevitable. Health services will need to be skilled, equipped and resourced to respond to this need.

## Figures and Tables

**Table:** 

Co-researchers	People either diagnosed and living with dementia or their carers
Facilitators/co-facilitators	NHS staff delivering the intervention
Interventionists	Group term for the facilitators/co-facilitators and supervisors who took part in the interviews
Supervisors	NHS staff supervising those delivering the intervention

## Data Sharing Statement

The datasets generated and analysed for this study will be available upon request from the corresponding author.
